# Impact of dietary changes on retinal neuronal plasticity in rodent models of physical and psychological trauma

**DOI:** 10.3389/fgene.2024.1373447

**Published:** 2024-09-13

**Authors:** Mital Y. Patel, Ruoting Yang, Nabarun Chakraborty, Stacy-Ann Miller, James C. DeMar, Andrew Batuure, Donna Wilder, Joseph Long, Rasha Hammamieh, Aarti Gautam

**Affiliations:** ^1^ TechWerks, Arlington, United States; ^2^ Medical Readiness Systems Biology Branch, Walter Reed Army Institute of Research, Silver Spring, MD, United States; ^3^ Blast-Induced Neurotrauma Branch, Walter Reed Army Institute of Research, Silver Spring, MD, United States

**Keywords:** blast injury, acute traumatic stress, omega-3 PUFAs, retinal transcriptomics, inflammation, neuronal plasticity

## Abstract

**Introduction:**

Blast injury has been implicated as the major cause of traumatic brain injury (TBI) and ocular system injury, in military operations in Iraq and Afghanistan. Soldiers exposed to traumatic stress also have undiagnosed, chronic vision problems. Here we hypothesize that excessive intake of ω-6 fatty acid linoleic acid (LA) and insufficiency of dietary long chain ω-3 polyunsaturated fatty acids (PUFAs, e.g., docosahexaenoic acid; DHA) would dysregulate endocannabinoid-mediated neuronal plasticity and immune response. The study objective was to determine the effect of blast-TBI and traumatic stress on retinal gene expression and assess the role of dietary deficiency of long chain ω-3 PUFAs on the vulnerability to these injury models.

**Methods:**

Linoleic acid was used as an independent variable to reflect the dietary increase in LA from 1 percent of energy (en%) to 8 en% present in the current western diets, and these custom LA diets were also devoid of long chain ω-3 PUFAs. Animals were exposed to a simulated blast overpressure wave followed by a weight drop head-concussion to induce TBI. A Separate group of rats were subjected to traumatic stress by a forced immersion underwater.

**Results:**

Our findings showed that blast-TBI exposure, post 14 days, produced significant neuropathological changes such as axonal degeneration in the brain optic tracts from all the three diet groups, especially in rats fed the DHA-deprived 1 en% LA diet. Transcriptomic analysis showed that presence of DHA in the house chow diet prevented blast-induced disruption of neuronal plasticity by activating molecular networks like SNARE signaling, endocannabinoid pathway, and synaptic long-term depression when compared to DHA-deprived 8 en% LA diet group. Under traumatic stress, retinal synaptic function, neurovascular coupling, and opioid signaling mechanisms were dysregulated in rodents fed DHA-deficient diets (i.e., 8 en% LA and 1 en% LA), where reducing the levels of ω-6 linoleic acid from 8 en% to 1 en% was associated with increased neuronal plasticity and suppressed immune signaling.

**Conclusion:**

The findings of our study suggest that deprivation of long chain ω-3 PUFAs in the diet affects endocannabinoid-mediated neuronal plasticity, vascular function and inflammatory response that could influence the resistance of veterans to TBI and psychological trauma.

## 1 Introduction

The high prevalence of mild traumatic brain injury (TBI) and traumatic stress is an imminent global health challenge and the predominant cause of long-term or permanent disability among military personnel (Ling et al., 2009). Distinguishing between the effects of TBI and traumatic stress on the sensory organs using a knowledge-driven unbiased panel of biomarker signatures will be essential for designing precise care management in Warfighters. Visual deficits are commonly reported among the victims of blast-induced TBI (Cockerham et al., 2011; Choi et al., 2015). It has been previously reported that more than 80% of service members experiencing TBI also develop symptoms of visual system injuries including visual field deficits, photophobia, reduced contrast sensitivity, impaired accommodation, and oculomotor dysfunction (Magone and Shin, 2014; Wang et al., 2014). Studies in rats have shown that exposure to single as well as repeated low-level blast overpressure induces cellular apoptosis and upregulates the expression of pro-inflammatory cytokines in the optic nerve (Choi et al., 2015). In a rat model of closed-head injury, stimulation of networks linked to release of prostaglandins were observed in the cerebellum and hippocampus, and they affected gene networks associated with membrane lipids, which could trigger axonal degeneration in the hippocampus (Chakraborty et al., 2021). Concussive events to the head alone following explosions could also be very detrimental to the eyes as it can result in degeneration of the brain visual processing centers and optic nerves leading to retrograde loss of retinal ganglion cells, retinal thinning, and retinal dysfunction. In particular, the axons within white matter tracts are more sensitive to injury caused by head trauma, even without any direct collateral damage to the eyes (Goodrich et al., 2007).

Besides disruption resulting from TBI, traumatic stress often leads to secondary perturbations in the brain and visual system. Previous studies have demonstrated positive correlation between severe stress and atrophy of the visual cortex and bilateral thalamus in an animal model (Yoshii et al., 2017). The effects of chronic restraint and emotional-pain stress on the dysregulation of visual evoked potentials have been shown in rat models, which were associated with increased oxidative stress in the retina and brain (Aydin et al., 2005; Shvedova et al., 1982). In the present study, traumatic stress was simulated using underwater trauma (UWT) model, which is widely used to produce anxiety-like behavior. UWT has a significant impact on neuronal activity and plasticity in the hippocampus and amygdala (Ardi et al., 2014; Kaur et al., 2020).

An imbalanced ratio of ω-3/ω-6 PUFAs (omega-3 and omega-6 polyunsaturated fatty acids) in modern western diets has been associated with a decreased proportion of long chain ω-3 PUFAs such as DHA and EPA (eicosapentaenoic acid) in the brain and retina. These long chain fatty acids serve as essential structural components of the central nervous system, and their deficiency leads to abrogation of endocannabinoid-mediated neuronal functions (Carrié et al., 2000; Lafourcade et al., 2011). Diets with high levels of long chain ω-3 PUFAs (ω3:ω6 = 7:1) and balanced supplements (ω3:ω6 = 1:1) have demonstrated potential benefits in improving the behavioral resilience to aggressor-exposed social stress in a mouse model. Moreover, transcriptomics data revealed that these balanced diets emerged favorable to brain development by inhibiting gene networks linked to neurodevelopment disorder, cell mortality and behavioral deficits post social stress (Chakraborty et al., 2023). Additionally, ω-3 PUFAs play an important role in neuroinflammation by converting into potent anti-inflammatory metabolites, e.g., resolvins, while ω-6 PUFAs like arachidonic acid gets metabolized into pro-inflammatory prostaglandins and leukotrienes. Numerous studies have reported correlation between DHA insufficiency and dysregulation in the structure and function of visual system, and the importance of long chain ω-3 PUFAs in the preservation of neuronal and phototransduction activities (Perumal et al., 2022; SanGiovanni and Chew, 2005). Hence, targeted dietary manipulation could be a promising approach for developing varying bioactive lipid autocoids posited to modulate ocular pathology in a traumatic model of physical and psychological injury. Our objective of the present study was two-fold; first, to determine the effect of military-relevant traumatic insults on transcriptomic changes and molecular signaling networks in the retina, and second, to compare the modulations of retinal gene expression following different dietary inputs of ω-3 and ω-6 PUFAs and deciphering its relationship to the ocular vulnerability, specifically to blast-TBI and traumatic stress.

## 2 Methods

All animal experiments were conducted in accordance with the Animal Welfare Act and other federal statutes and regulations relating to animals and experiments involving animals and adhered to principles stated in the Guide for the Care and Use of Laboratory Animals (NRC Publication, 2011 edition) using an Institutional Animal Care and Use Committee-approved protocol. Male Sprague Dawley rats, 8–9 weeks old that weighed 270–290 g (Charles River Laboratories, Wilmington, MA) were housed at 20°C–22°C (12 h light/dark cycle) with free access to food and water ad libitum.

### 2.1 Subjects and Diets

In our study, rats were fed on one of three isocaloric custom-made rodent chows (Dyets Inc., Bethlehem, PA). 1. 1 en% LA diet: 1% of caloric energy (en%) as derived from linoleic acid (LA; ω-6 PUFA) with 1 en% as α-linolenic acid (LNA; ω-3 PUFA); (Alvheim et al., 2012), 2. 8 en% LA diet: 8 en% as LA with 1 en% as α-LNA. 3. HC diet: NIH approved animal-facility house chow (HC) diet, well- balanced in all PUFAs (ω-6, ω-3, EPA, DPA, and DHA). The animals are then exposed to a TBI event from high fidelity blast waves generated in an advanced blast simulator (ABS) and a weight drop skull concussion (Marmarou et al., 1994) or an underwater trauma (UWT) stressor (Moore et al., 2012). Both TBI and UWT exposed animals were fed three different diets for 6 weeks prior to insult and continued thereafter. Shams only received handling and/or anesthesia procedures.

### 2.2 Blast-TBI Model

A group of rats (n = 6) was subjected to a closed-head TBI model consisting of blast overpressure (BOP) wave exposure coupled with weight drop concussion (i.e., Marmarou method) (Marmarou et al., 1994). This two-hit model simulates the occurrence of a primary blast injury (shock wave) followed by a secondary one (thrown object). The rats were anesthetized with 5% isoflurane for 3 min. They were then secured in the Advanced Blast Simulator (ABS) shock tube and exposed once to a BOP wave (20 psi), which was immediately followed by dropping a 500-gram metal weight from 125 cm above onto a stainless-steel disc surgically affixed to the rat’s skull midway between lambda and bregma, whereas shams (SH; n = 6) received anesthesia alone (Marmarou et al., 1994). No analgesics were used other than topical lidocaine on the surgical site. At 14 days post-insult, the rats were euthanized using 4%–5% isoflurane followed by blood exsanguination using cardiac puncture. The animal was then decapitated to finish the euthanasia, and aid removal of the tissues.

### 2.3 Underwater Trauma

In parallel, a group of rats (n = 6) was subjected to a UWT stressor model (i.e., Richter-Levin method) (Richter-Levin, 1998) that consisted of 30 s of swimming in a tank of room temperature normal-saline and habituation, followed by 30 s of forced whole body immersion using a plunger device. Shams received 1 min of free swimming (SH). The animals were then euthanized using the same procedure mentioned above, at 14 days post-exposure to traumatic stress.

### 2.4 Histopathology for Brain Optic Tracts of Blasted-Rats

Rats from the house chow and DHA-deficient diet groups were euthanized at 14 days post-blast exposure for histopathology assessments of their brain visual centers. Animals were deeply anesthetized by isoflurane inhalation. After surgical opening of the chest and insertion of gravity flow lines in the heart, the animals were perfused transcardially with physiological saline, which results in euthanasia by blood exsanguination, followed by phosphate buffered 4% paraformaldehyde saturated with picric acid (FD Neurotechnologies, Inc.) to fix the tissues.

The brain optical tracts were carefully dissected from the paraformaldehyde-perfused rat heads, and fixation of brains was continued by immersion for up to 6 h in phosphate buffered 4% paraformaldehyde saturated with picric acid, and then washed overnight with buffered 20% sucrose solution. Fixed brain regions were sectioned, stained, and mounted on microscope slides by FD Neurotechnologies, Inc. Brains were cut into serial coronal sections (30–50 µm) through the cerebrum at 11 evenly-spaced positions from stereotaxic coordinates of bregma 1.0 mm to −8.3 mm, as mounted in triplicate. The brain sections target all major visual centers. Slides were prepared for the tissue sections that underwent silver impregnation. The resulting silver stain is highly reactive to disrupted proteins which highlight as a brown to black color within axonal fiber tracts, revealing degeneration by differences in morphology and staining intensity. For all prepared slides, mosaic (12 × 14) pictures of the brain regions of interest (e.g., optic tracts) were taken using an Olympus BX61 microscope (Olympus Corporation, Center Valley, PA, United States) and Stereo Investigator tool (MBF Biosciences, Williston, VT, United States). The intensity of the silver staining in the captured images was quantified using densitometry (lumens/mm^2^) as assessed by Image-Pro Premier software (Media Cybernetics Inc., Rockville, MD, United States).

### 2.5 Tissue collection and transcriptomic assay

Fourteen days (14 days) post-traumatic insult, animals were euthanized by deep isoflurane anesthesia and exsanguination via cardiac puncture for removal of tissues and the collection of blood. The retina tissue was micro-dissected from the enucleated eyes (after removal of the cornea, lens, and vitreous), rinsed in ice cold RNAlater^®^ solution (Invitrogen, Thermo Fisher Scientific, Waltham, MA), and then snap frozen in liquid nitrogen, prior to storage at −80°C until RNA extraction. Total RNA was isolated from homogenized retinas using a hybrid protocol that utilized both TRIzol™ reagent (Invitrogen) and Qiagen’s miRNeasy Mini kit (Qiagen, MD). The manufacturer’s protocol for TRIzol™ reagent was followed until the separation phase, i.e., 1.5 volumes of ethanol were added to the aqueous extract, and the RNeasy Mini columns were used to elute the total RNA per Qiagen’s instructions. RNA purity and concentration were evaluated using a NanoDrop™ 2000 spectrophotometer (Thermo Fisher Scientific). The RNA quality was assessed as RNA integrity number (RINs) on Agilent Tapestation 2,200 platform (Agilent Research Laboratories, Santa Clara, CA). Most RIN values were larger than 7.0, and samples with low RIN value had been removed from the study. Expression microarrays were performed using Rat Gene Expression Microarray Kits (GPL14797: Agilent-028279 SurePrint G3 Rat GE 8 × 60K Microarray) following the manufacturer’s protocol (Agilent Research Laboratories, Santa Clara, CA) and our previously published method (Gautam et al., 2015). These slides contain 62,976 probes that include 52,563 probes featured with at least one genome alignment and represent 16,080 distinct rat genes. Cy-5 labeled 200 ng of purified RNA was co-hybridized with a Cy-3 labeled reference RNA purchased from Agilent Technologies, Inc., CA, United States of America. The specific activity values used for Cy-5 and Cy-3 were 6 and above. Post overnight hybridization at 65°C, the slides underwent a series of washes to complete the process. Hybridized microarray slides were scanned using Agilent’s G2600D-Scanner, and images were processed using the default set-up of Agilent’s Feature Extraction Software v12.0.3.1. The raw files have been assigned to accession number GSE243996 at GEO database.

### 2.6 Statistical analysis

The gene expression profile was obtained using Agilent’s Feature Extraction Software v10.7. Data normalization was achieved through the Locally Weighted Scatterplot Smoothing (LOWESS) algorithm using the Limma R package (Smyth, 2005). A couple of outliers were removed via principal component analysis. Differential analysis was conducted using the Limma moderated pair wise t-test with an alpha level of 0.05. The resulting differentially expressed genes (DEGs) were then submitted to the Ingenuity Pathways Analysis platform (QIAGEN Inc., Valencia, CA) for pathway and network enrichment. Enriched pathways had to meet criteria including a hypergeometric test *p*-value <0.05, more than five DEGs, and a non-zero enrichment Z-score. Pathways with positive Z-scores were considered activated, while those with negative Z-scores were designated as inhibited pathways. Statistical analyses were visualized using GraphPad^®^ version 9.0 software (GraphPad Software Inc., La Jolla, CA). Experimental results were expressed as mean ± standard error of the mean (SEM), and unpaired t-tests were used to assess phenotypic shifts at an alpha level of 0.05.

## 3 Results

### 3.1 Blast exposure led to axonal degeneration of brain optic tracts in rats fed house chow and 1 and 8 en% LA diets at 14 days post-insult

**FIGURE 1:**
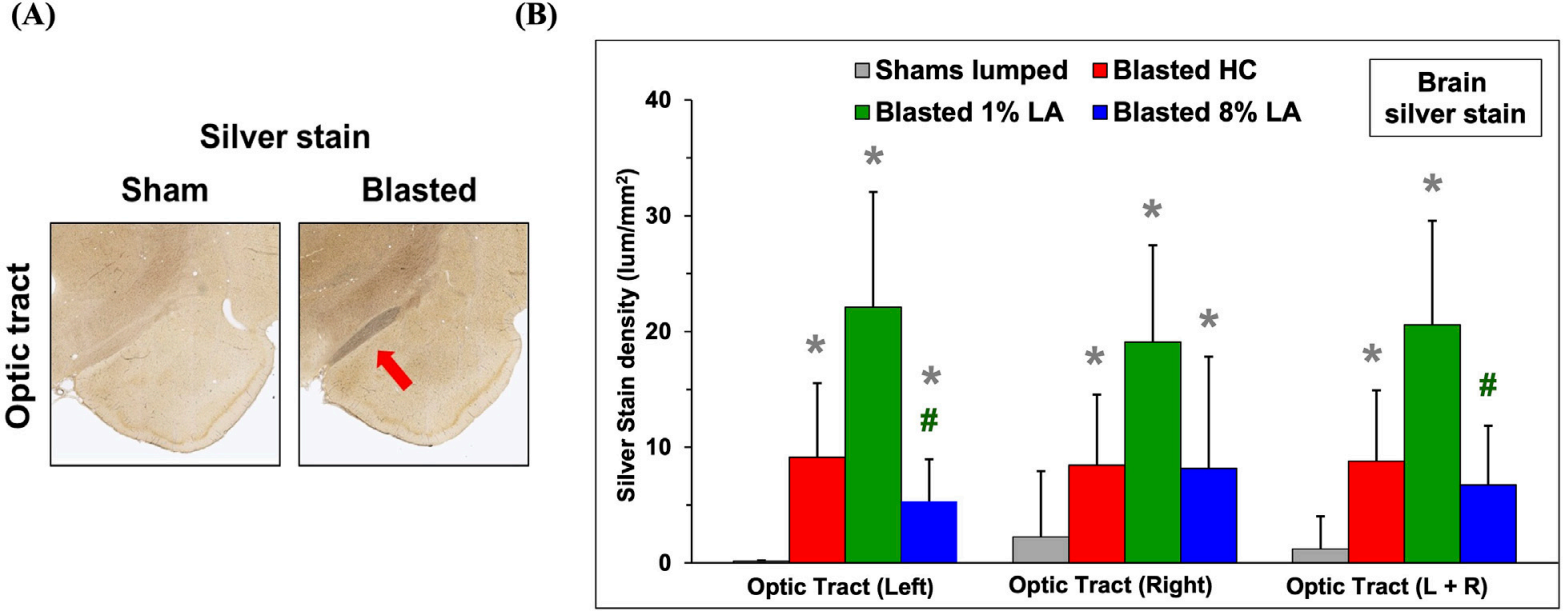
**(A)** Representative histopathological images (10x) are displayed for the interconnecting left side brain optic tracts from the corresponding sham and blasted animals. Silver stains highlight axonal fiber degeneration in the optic tracts that lie directly between the hypothalamus and amygdala regions of the rat brain. **(B)** Bar graphs depicting silver stain density of brain regions from sham (lumped) and blast plus weight drop exposed rats at 14 days post-insult (mean ± SD, n = 4). Brain optic tract images were taken at a higher power magnification to help reveal cellular feature. *p ≤ 0.05; significant difference from shams, as by t-test. #*p* ≤ 0.05; significant difference between diets.

The histopathology of brain optic tracts was performed by silver staining at 14 days following TBI. Shown in Figure 1A are representative images of the brain sections for corresponding sham and blasted rats from the house chow diet group, at 2–20× magnification of the left optic tract and associated brain regions. The left optic tract is innervated with the right retina through substantial axonal cross over (>20%) at the optic chiasm. The blasted rats appear to have ongoing axonal fiber tract degeneration in their left optic tracts when compared to shams. The right optic tracts also had signs of neurodegeneration (images not shown). Also shown is a bar graph for the silver stain densities in the brain optic tracts at 14 days post-blast; and in this case, the shams are all the animals combined for each diet (lumped shams = grey, HC = red, 1 en% LA = green, and 8 en% LA = blue, n = 4). Overall, these results demonstrate that the effect of blast on axonal degeneration was much higher in DHA-deficient 1% versus 8 en% LA diet group (Figure 1B).

### 3.2 Dysregulation of retinal gene expression on exposure to blast-TBI or traumatic stress

**FIGURE 2:**
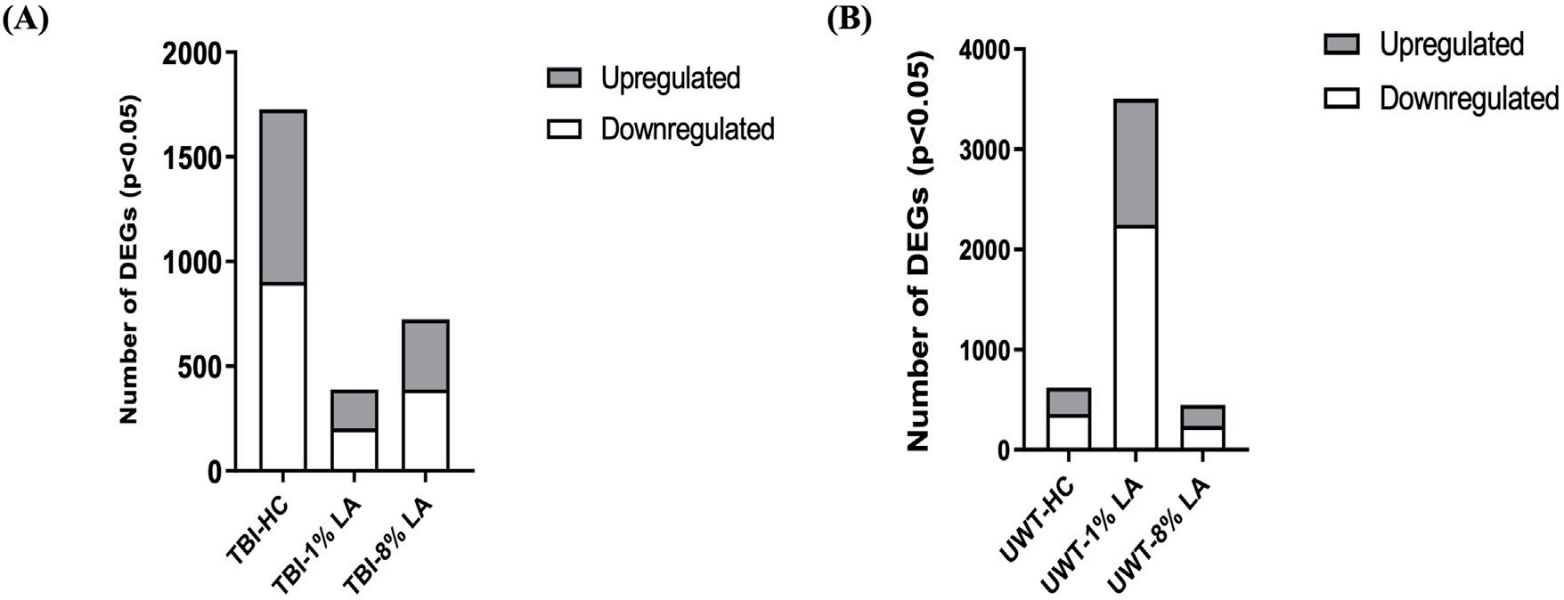
**(A)** Blast plus head concussion effects on retinal gene expression in three different diet groups: TBI-HC, TBI-8 en% LA and TBI-1 en% LA. **(B)** The number of retinal DEGs in response to traumatic stress under different dietary conditions: UWT-HC, UWT-8 en% LA and UWT-1 en% LA. Up- (gray) and downregulated (white) genes are indicated on the side of the graph (*p* < 0.05).

Analysis of differentially expressed genes (DEGs) in the rat retinal tissue in response to traumatic insults was undertaken using whole-genome rat microarrays, as described above. The animals in the blast-TBI study were defined as three groups: animals fed with house chow diet (TBI-HC), 8 en% LA diet (TBI-8% LA) and 1 en% LA diet (TBI-1% LA). We compared the gene expression between TBI and Sham rats in each diet groups. At 14 days post-TBI, we observed 335 upregulated and 388 downregulated DEGs in 8% diet group, and showed 186 up- and 203 downregulation in 1 en% LA diet when compared to a much higher 825 up- and 902 downregulation in HC diet (Figure 2A). Nearly 110 DEGs overlapped between HC and 8 en% LA diet (Supplementary Figure S1A), while only 38 were common between HC and 1 en% LA diet, including DEGs involved in mitochondrial biogenesis (ACAD10), serotonergic neurotransmission (HTR3A), synaptic activity (EGR1 and P2RX2), and interferon signaling (IFI2712B and IRF7) (Supplementary Figure S1A; Supplementary Table S1).

To assess the disparity in gene expression occurring at 14 days after exposure of the animal to an underwater trauma stressor event, which can systemically alter the physiology of the eye, the stress-specific array dataset was compared with a dataset from sham animals. The 1 en% LA diet group has several times more DEGs than the other groups (Figure 2B). Seventy-two out of the 113 common DEGs between 8 en% LA and 1 en% LA diet had opposite regulation direction (Supplementary Figure S1B; Supplementary Table S4). Among the 72 genes, we found that the expression of neuronal genes associated with nociception (OPRL1), excitotoxicity (GRIA2 and ARHGEF9), and behavioral locomotion and anxiety (HTR1D) were upregulated under psychological stress in the 1 en% LA diet animals, while downregulated in the 8 en% LA diet group. In contrast, high levels of ω-6 PUFA in the 8 en% LA diet potentially led to upregulation of inflammatory genes, including PTGS1, TNFRSF26, and CASP8 (Supplementary Table S4). The fact that there are relatively higher number of dysregulated genes in the 1 en% LA diet suggests that the transcriptomic changes following traumatic stress are dependent upon both decreases in ω-3 and ω-6 PUFA availability.

### 3.3 Nutritional restriction of ω-3 DHA changes the pattern of neuronal, vascular, and immune networks in the retina on exposure to blast-TBI

**FIGURE 3:**
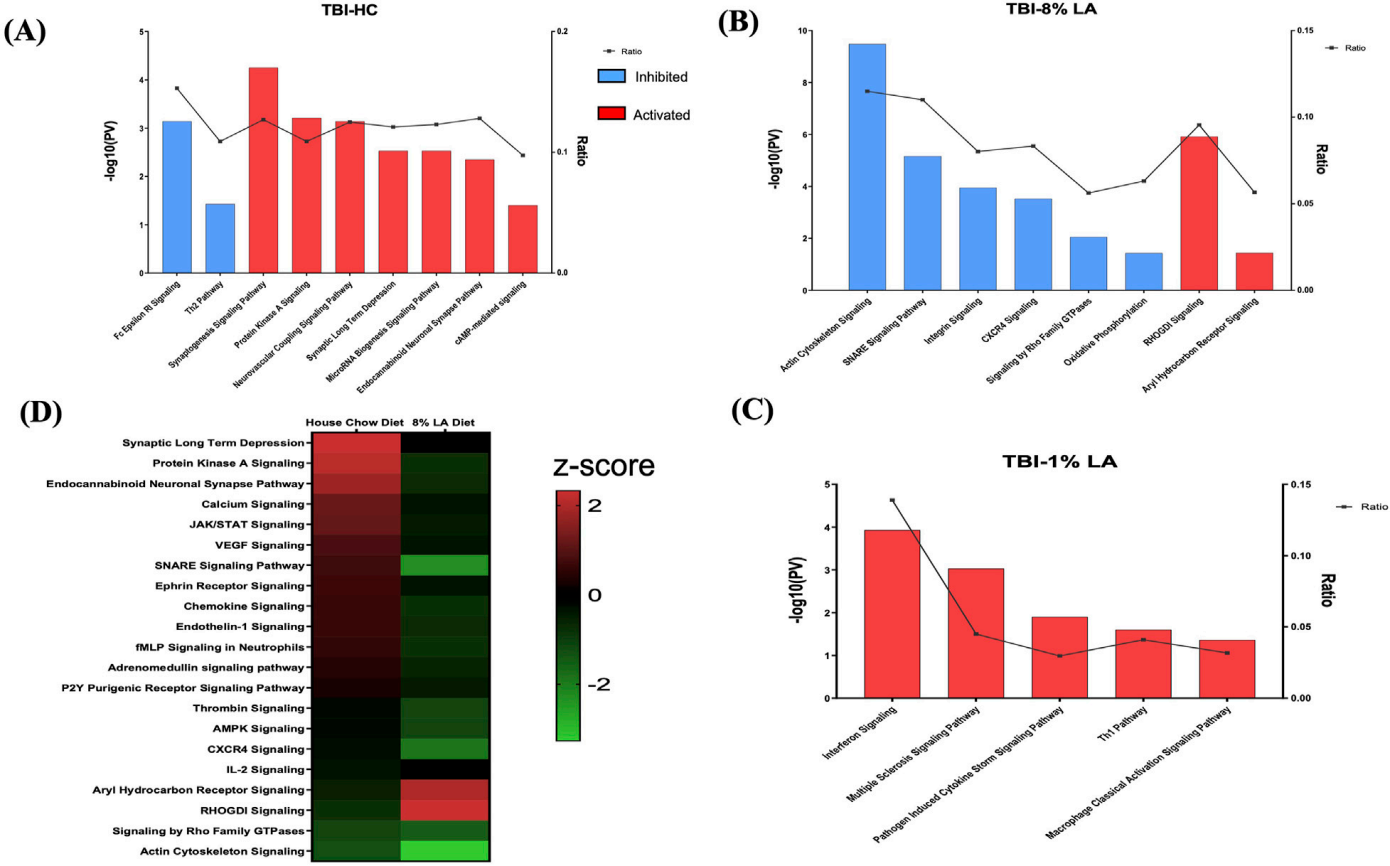
Bar graph shows signaling pathways dysregulated in the retina in response to blast overpressure plus head concussion under different dietary conditions. **(A)** TBI and house chow diet **(B)** TBI and 8 en% LA diet, and **(C)** TBI and 1 en% LA diet (pv < 0.05 and |z-score| > 1.5), with the x-axis representing the signaling pathway and the *y*-axis representing the -log10 *p*-value. The ratio indicates the percentage of DEGs detected in that particular pathway under the influence of TBI and dietary conditions. Molecular networks with z-scores greater than 1.5 were considered activated (red), while those with z-scores lower than −1.5 were designated as inhibited (blue) pathways. **(D)** The heatmap displays the pathways that are commonly enriched between DHA-rich (House chow) and -deficient (8 en% LA) diet (pv < 0.05). The red/green color stands for the activated/inhibited pathways as compared to respective shams.

Functional analysis was carried out using the genes listed under TBI-HC, TBI-8 en% LA and TBI-1 en% LA, respectively, to determine the effect of dietary modifications on TBI-induced disruption of biological and canonical pathways in the retina. The findings from our study showed that altering a diet’s content of ω-3 PUFAs was able to influence the ocular molecular networks in response to physical injury induced by blast overpressure waves and head concussion. The overall number of dysregulated networks in the retina was higher for the TBI-HC diet group (98 as compared to 47 networks for TBI-8 en% LA and 9 networks for TBI-1 en% LA group), as described in Supplementary Tables S2–S3 (*p* < 0.05). The list was filtered (|z-score | > 1.5; *p* < 0.05) to include those molecular pathways that were significantly enriched and physiologically relevant to ocular immunogenicity, as well as the perseverance of neuronal and synaptic integrity (Figure 3). Macrophage classical activation pathway, interferon signaling, Th1/Th2 pathways, and CXCR4 signaling, among others, emerged as the major networks associated with immunological function in the retina.

Considering the ability of DHA to accumulate and be avidly retained in the phospholipids constituting the central nervous system (CNS) membranes (primarily synapses, dendrites, and photoreceptor outer segments) (Bazan et al., 2011), understanding the role of this fatty acid in neuroprotection, memory, and vision becomes of utmost importance in TBI models. Dietary inclusion of DHA (i.e., HC diet) stimulated synaptogenesis signaling and neurovascular coupling mechanisms (Figure 3A; Supplementary Table S2), thus exhibiting a potential neuroprotective effect against ocular trauma in rats exposed to blast injury. A striking observation in this ω-3 DHA enriched diet was the activation of microRNA biogenesis signaling pathway in response to traumatic injury (Figure 3A; Supplementary Table S2). Group comparisons of the HC and 8 en% LA (i.e., DHA deprived) diets showed that the former significantly ameliorated synaptogenesis and neural membrane biogenesis in response to TBI, as demonstrated by activation of SNARE signaling, endocannabinoid neuronal synapse pathway, and ephrin receptor signaling (Figure 3D). Other biological processes that are differentially regulated between these diets include AHR (aryl hydrocarbon receptor) signaling, calcium regulation and protein kinase A signaling. The DHA-enriched HC diet activated adrenomedullin and VEGF (Vascular endothelial growth factor) signaling mechanisms while an opposing effect was observed with the DHA-deprived 8 en% LA diet (Figure 3D). This may indicate that long chain ω-3 PUFAs have protective effect on vascular function, angiogenesis, and neurogenesis. A total of 21 networks were commonly enriched for TBI-HC and TBI-8 en% LA, while none were observed between 1 en% LA and 8 en% LA diet or 1 en% LA and HC diet.

Furthermore, the rats receiving dietary DHA showed less ocular inflammation after TBI as demonstrated by reduced activity of the pro-inflammatory cytokines IL-8, IL-6, and IL-2, and suppressed ferroptosis signaling and senescence pathway in the retina (Supplementary Table S2). Together, the results of the pathway analysis indicate that the presence of DHA in the diet diminishes cell death and inflammatory signaling while positively influencing neuronal and vascular integrity within the retina of animals exposed to blast-TBI.

### 3.4 Traumatic stress affects distinct molecular pathways in the DHA-deficient groups

**FIGURE 4:**
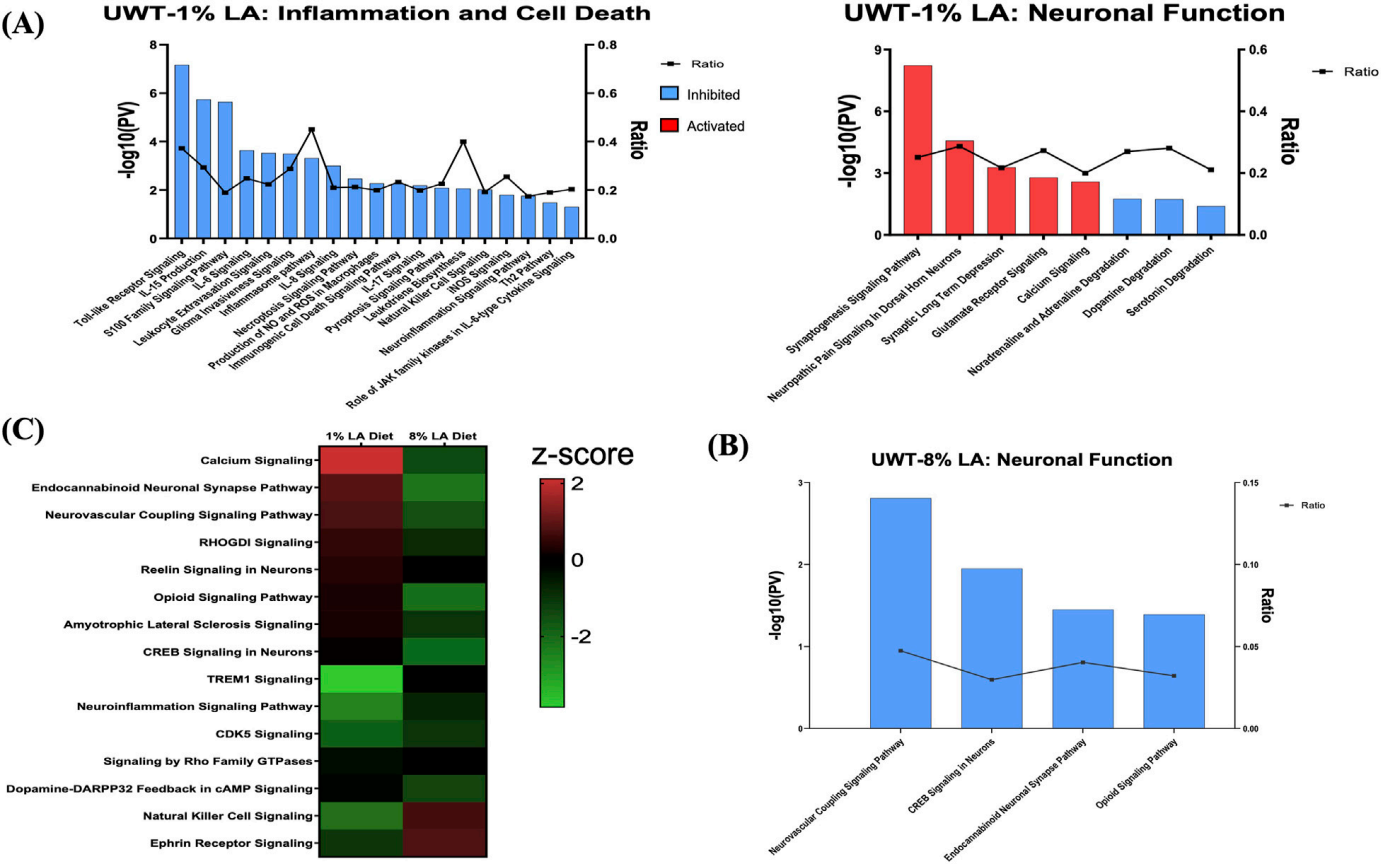
**(A)** Bar graph showing the effect of traumatic stress and 1 en% LA diet on the inflammatory and cell death pathways (left panel), and neuronal signaling mechanisms (right panel) in the retina. **(B)** Influence of traumatic stress and 8% diet on ocular neuronal function (pv < 0.05 and |z-score| > 1.5), with the x-axis representing the signaling pathway and the *y*-axis representing the -log10 *p*-value. The ratio indicates the percentage of DEGs detected in that particular pathway under the influence of traumatic stress and dietary conditions. Signaling pathways with z-score >1.5 were considered activated (red), whereas those with z-score < −1.5 were designated as inhibited (blue) pathways. **(C)** Heatmap showing the common molecular networks between 1% diet and 8% diet (pv < 0.05), and the pathway activity is represented by the z-score. The red/green color stands for the activated/inhibited pathways as compared to respective shams.

To determine the role of dietary changes on different biological pathways following the traumatic stress insult, we performed ingenuity pathway analysis on the transcriptomic changes observed in each of the three respective treatment groups. Figure 4A enlists ocular signaling networks in the 1 en% LA group. Interestingly, inflammatory responses such as those associated with TLR (Toll-like receptor) signaling, iNOS signaling, S100 family, and production and activity of pro-inflammatory cytokines and leukotrienes were shown to be suppressed under traumatic stress in the animals fed with the 1 en% LA diet (Figure 4A, left panel). The inflammatory proteins such as iNOS and S100B are known to play a vital role in neurological disorders including epilepsy, schizophrenia and TBI (Langeh and Singh, 2021; Hong et al., 2016). Our results also demonstrated an increase in the expression of genes associated with the neurotransmitter systems such as serotonin, glutamate and sympathomimetics (i.e., adrenaline, noradrenaline, and dopamine), as well as synaptic plasticity in the retina, observed at 14 days post-stress (Figure 4A, right panel; Supplementary Table S5). Other stress-induced changes in ocular gene expression are related to oxidative stress (e.g., PXR and AHR signaling; Supplementary Figure S2A), and GPCR, MAPK and aldosterone signaling (Supplementary Figure S2B; Supplementary Table S5).

Fifteen pathways were commonly enriched for the 1% and 8 en% LA diets, while UWT stressor seemed to have opposing effects on neurological functions between them. 8 en% LA cohorts have significant downregulation of neurovascular coupling, endocannabinoid neuronal synapse pathway, opioid, and CREB signaling (Figures 4B, C; Supplementary Table S6A). On the contrary, the traumatic stress insult did not have a significant effect on retinal signaling in rats fed the DHA-enriched HC diet other than the attenuation of dopamine-DARPP32 feedback in cAMP signaling and endocannabinoid neuronal synapse pathway, and activation of anti-inflammatory IL-10 signaling (Supplementary Table S6B).

### 3.5 Common neuronal and non-neuronal signaling networks altered across different diet groups and traumatic insults

**FIGURE 5:**
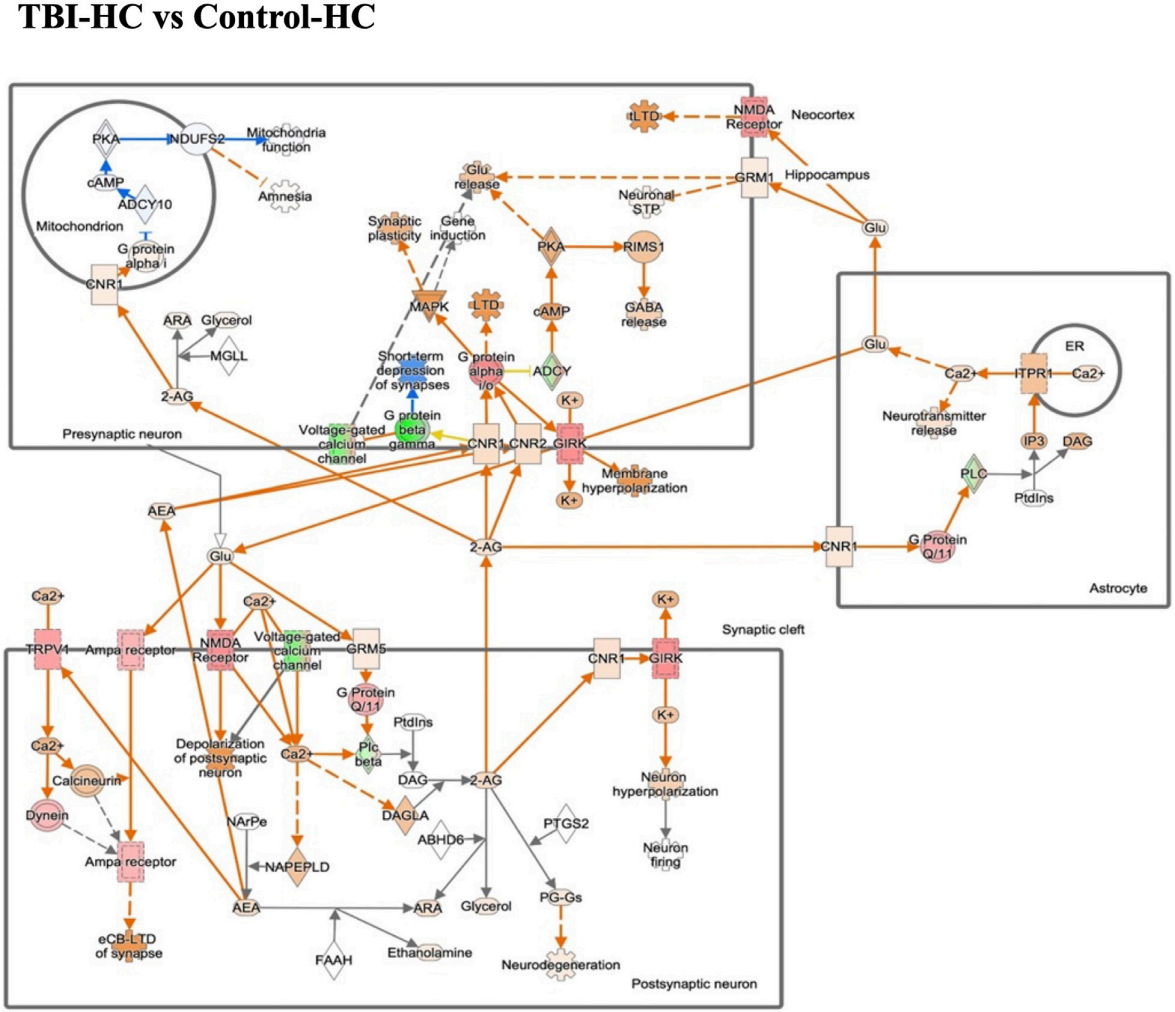
The gene expression in endocannabinoid neuronal synapse pathway in the blast house chow group compared with sham house chow group. Endocannabinoids regulate synaptic function through retrograde signaling, autocrine signaling, and also indirectly by activating astrocytic receptors. Green/Red means down-/up-regulation. Blue network means inhibition while orange means activation.

Considering all of the ocular pathways identified as hits in the different diet and insult groups, the one that was significantly altered in expression in most categories is the endocannabinoid neuronal synapse pathway. Interestingly, retinal endocannabinoid signaling was shown to be inhibited in the 8 en% LA diet group after TBI when compared to the DHA enriched HC cohort. The dysregulated genes in endocannabinoid neuronal synapse pathway are focused on various receptors and ion channels (Figure 5). For the traumatic stress study, this neuronal network was attenuated in animals fed with diets having a higher ω-6: ω-3 ratio (i.e., House Chow and 8 en% LA diet), with greater inhibitory effect shown in the DHA-deficient 8 en% LA cohort (Supplementary Tables S6A, B). In contrast, for the balanced 1 en% LA diet group, the ocular endocannabinoid signaling was significantly activated after traumatic stress exposure (Supplementary Table S5; Supplementary Figure S3). Additionally, other molecular pathways that were differentially regulated in the 8 en% LA diet group on exposure to traumatic stress or blast-TBI include calcium and RHOGDI signaling (Supplementary Tables S7A–C). In the 1 en% LA cohort, both traumatic stress and TBI had different effects on the immune networks such as Th1 and pathogen induced cytokine storm signaling pathway as well as on the regulation of the epithelial mesenchymal transition by growth factors (Supplementary Tables S8A–C).

**FIGURE 6:**
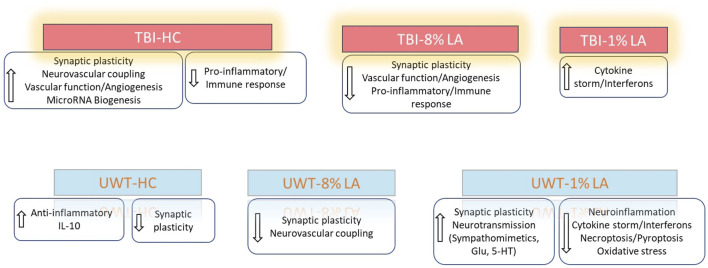
Summarizes the influence of DHA-rich and -deficient diets on the ocular neuronal plasticity, inflammatory response and vascular function in the rats exposed to different traumatic events.

To summarize, our results demonstrated that exposure to blast-TBI disrupted retinal neuronal plasticity and vascular function in rats consuming 8 en% LA diet while it increased the ocular inflammatory response in the 1 en% LA group. Induction of traumatic stress in the DHA-deficient 1 en% LA group dysregulated the ocular transcript profiles such as those associated with activity of neurotransmitters like sympathomimetics and glutamate as well as suppressed inflammation-induced cell death mechanisms in the retina (Figure 6).

## 4 Discussion

The present study investigated effects of blast-TBI or traumatic stress on retinal gene expression patterns under the influence of different dietary conditions. Converging an important functional network from thousands of genes provided knowledge on the pathways involved in the role of ω-3 and ω-6 PUFAs in ocular injury induced by physical and psychological trauma.

### 4.1 Ocular blast injury disrupts synaptic and vascular homeostasis associated with dietary deficiency of ω-3 DHA in the high-fat omega-6 diet

Studies have reported the influence of blast overpressure on ocular neuroinflammation and cell death in animal models (Mammadova et al., 2017). Blast exposure can lead to structural damage of the cerebral vasculature, especially at the pericyte - endothelial cell junctions within capillaries, followed by disruption of gliovascular and neurovascular interactions and thus affecting cerebral blood flow (Abutarboush et al., 2019; Gama Sosa et al., 2014; Gama Sosa et al., 2019).

Long chain ω-3 PUFAs such as DHA are known to play an essential role as bioactive molecules in synaptic formation and plasticity, and help to maintain neuronal membrane stability (Zárate et al., 2017). Low levels of DHA in the CNS are associated with an increased astrocyte reactivity and microglial activation that interferes with the correct pattern of neural connectivity (Madore et al., 2014; Sandre et al., 2021). TBI leads to disruption of hippocampus SNARE complex (Carlson et al., 2016), an essential component of synaptic vesicle exocytosis involved in synaptic long term depression via release of L-glutamate, which is also the primary neurotransmitter produced by photoreceptor cells (Guoping et al., 2017; Yang, 2004). Our data showed that dietary inclusion of DHA suppresses such impairments in retinal synaptogenesis and SNARE complex formation induced by blast-TBI by stimulating the expression of synaptic genes such as SNAP91, SYN1, SYT10, SYT3 and SYT6. On the contrary, lack of dietary DHA as in 8 en% LA group abrogated the recovery of animals from TBI-induced deterioration of synaptic plasticity in the retina due to reduced expression of synaptic genes like SNAP25 and SYT1. Our transcriptomic results suggest that dietary inclusion of DHA suppressed blast-induced pro-inflammatory cytokine networks such as IL-8, IL-6, and IL-2, and ferroptosis signaling pathway. Based on the silver staining results, the impact of blast waves on axonal degeneration in the optic tracts was particularly high in animals fed DHA-deprived 1 en% LA diet indicating greater loss of neuronal connectivity within the retina.

In response to blast exposure, dietary inclusion of DHA led to stimulation of the retinal neurovascular coupling signaling pathway, that which been shown to occur as a result of activation of glutamate receptors both on neurons and astrocytes (Jessen et al., 2015; Naganawa and Taoka, 2022) and either directly or indirectly mediate the release of vasodilators (Attwell et al., 2010). One such molecular network regulating blood flow and promoting vascularization development in retina is the adrenomedullin signaling pathway (Attwell et al., 2010; Blom et al., 2012; Hirabayashi et al., 2019; Rey-Funes et al., 2013). Our results indicate that inclusion of DHA in the HC diet stimulates ocular adrenomedullin signaling in response to blast exposure, and possibly prevents TBI-induced disruption of blood flow within the retina. Additional aspects of vascular network such as VEGF and protein kinase A were also possibly activated by presence of dietary long chain ω-3 PUFAs post-exposure to the blast-induced ocular injuries.

**FIGURE 7:**
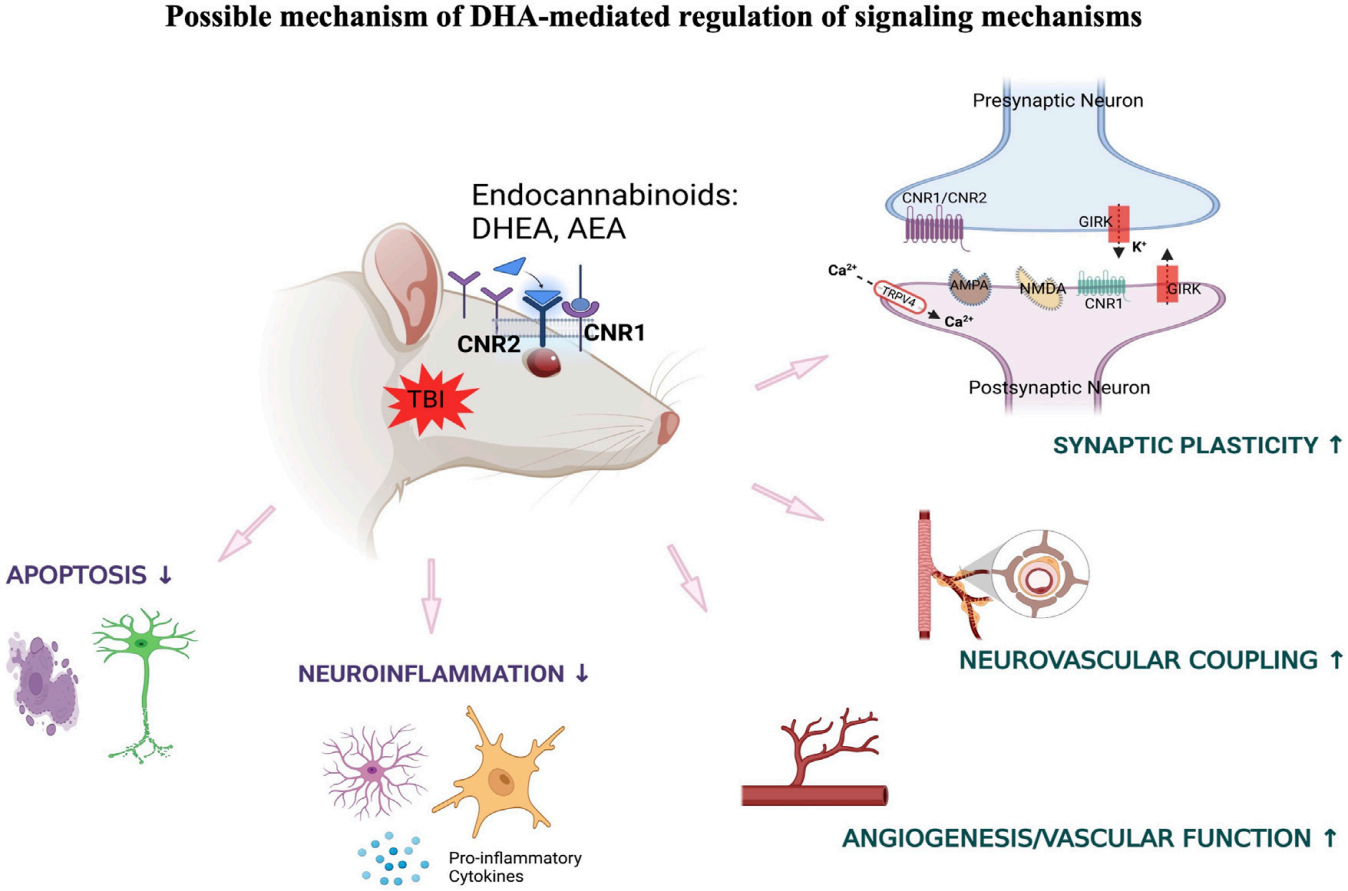
DHA-induced activation of endocannabinoid-mediated synaptic plasticity in response to TBI.

One likely mechanism for the involvement of long chain ω-3 PUFAs in regulation of synaptic function is its role in modulating the endocannabinoid system. Previous studies have demonstrated that dietary deprivation of ω-3 PUFA is associated with disruption of endocannabinoid-dependent synaptic function in different brain regions including prefrontal cortex and nucleus accumbens (Lafourcade et al., 2011; Larrieu et al., 2012). In this context, current data indicates that DHA mediates stimulation of ocular endocannabinoid receptors that may ameliorate synaptic plasticity and neurovascular coupling through various signaling mechanisms under traumatic conditions. Increased dietary intake of DHA has also shown beneficial effects towards blast-induced inflammatory and neurodegenerative processes, and this may also be potentially mediated through the activity of ω-3 endocannabinoids in the retina (Figure 7).

### 4.2 Targeted dietary manipulation influences the regulation of traumatic stress-induced retinal signaling involved in neurotransmission and inflammatory response

Diet-induced alterations in ω-3 and ω-6 fatty acids can alter the levels of lipid mediators such as endocannabinoids, and these changes are associated with a wide range of pathological conditions. Once the ratios between arachidonic acid and DHA changes in the organism it can lead to neurological diseases like depression, schizophrenia, or neurodegenerative pathologies (Bazinet and Layé, 2014; Joffre et al., 2014). In chronic headache patients, it has been shown that reductions in physical pain and psychological distress are correlated with increases in the endocannabinoid derivatives of ω-3 PUFAs, DHA, but not those from arachidonic acid (i.e., ω-6 PUFA) (Ramsden et al., 2015). A diet rich in ω-3 PUFAs had a positive impact on endocannabinoid plasticity in the brain (Lafourcade et al., 2011; Larrieu et al., 2012; Pan et al., 2011; Watanabe et al., 2003; Yamada et al., 2014).

In our study, we observed that animals fed with a 1 en% LA diet having a close balance with ω-3 PUFA (i.e., α-LNA) showed amelioration of endocannabinoid neuronal synapse signaling in the retina on exposure to stress while the regulation of ocular endocannabinoid plasticity was impaired in the rats fed with more imbalanced 8 en% LA diet. This suggests that, in comparison to control animals, induction of traumatic stress would possibly enhance the enzymatic metabolism of α-LNA in the 1 en% LA diet to DHA and/or EPA and further drive the synthesis of ω-3 derived endocannabinoids in the retina, with an essential role in synaptic function and neurotransmission. In support of this, we also saw a rise in the gene expression levels of serotonin and sympathomimetics in the 1 en% LA group, indicating stress-induced increase in adrenaline/nor-adrenaline and dopamine response along with glutamate receptor activity in the retina. The activity of DHA on the photoreceptors is directly controlled by adjacent retinal pigment epithelial (RPE) cells, which utilizes a specific DHA-phospholipid pool also as a precursor for the biosynthesis of inflammation resolving neuroprotectin D1 (NPD1) (Calandria et al., 2009). Both DHA and its metabolite, NPD1 are known to be involved in regulating the spread of seizures and decreasing its severity by modulating glutamate availability at the synapses through glutamate transporters (Berry et al., 2005; Musto et al., 2011). An increase in the activity of glutamate receptors both on neurons and astrocytes is also known to be associated with neurovascular coupling mechanisms mediated through transmission of direct and indirect vasoactive signals for the regulation of cerebral blood flow (Attwell et al., 2010). Previous studies have shown that long chain ω-3 PUFAs like DHA potentiates dopaminergic receptor activity (Jobin et al., 2023) and the importance of ω-6: ω-3 balance on depression pathophysiology through effects on the dopaminergic system (Sublette et al., 2014). Likewise, when compared to animals fed with 1 en% LA diet, the higher levels of ω-6 PUFA in 8 en% LA diet as well as a greater ratio to ω-3 PUFA, possibly contributed to impairment of certain neurotransmitter functions in the retinal neurons, such as the opioid, CREB and neurovascular coupling signaling pathway in response to acute traumatic stress.

Among other functions, the endocannabinoids are known to play a pivotal role in the regulation of local inflammatory state as well as immune response in the ocular tissues (Lafreniere and Kelly, 2018). An interesting finding in this study was a net anti-inflammatory effect exhibited by the 1 en% LA diet in the retina on exposure to stress, possibly mediated by endocannabinoids or α-LNA alone through stimulation of the PPAR (Peroxisome proliferator-activated receptors) transcription factors. By acting as agonists for sites on both PPARα and PPARγ, which then stimulate expression of the repressor protein PTEN (Phosphatase-tensin homolog), these lipid mediators negatively interfere through the PI3K/AKT pathway with NF-κB signaling, and its downstream pro-inflammatory cytokine networks (Figure 4A; Supplementary Figure S2A) (Daynes and Jones, 2002; Fezza et al., 2014). Thus, retinal tissue could be protected from stress-induced deterioration through the biosynthesis of endocannabinoids derived from ω-3 PUFAs in 1 en% LA diet with potent anti-inflammatory and anti-apoptotic effects. Overall, the current data strongly suggest that a dietary deficiency of retinal DHA would disrupt the correct pattern of neural connectivity under stressful conditions, through mechanisms related to endocannabinoid signaling.

Limitations of our study include the following: 1) The sample size of our treatment groups (n = 6) were relatively small; and thus, there was some indication that the study was slightly underpowered. For example, if we had increased the number of animals by 2-fold, the numerous borderline differences that we observed in the expressed genes (i.e., *p* = 0.04–0.05, ∼30% of total reported) may have been even further strengthened in significance. 2) The present work did not conduct any studies assessing retinal physiological function; however, we could have tried techniques like electroretinography (ERG) for photoreceptor light-stimulation response and optokinetics for assessing visual acuity. We could have then looked for any correlations between the retinal signaling pathway changes identified and physiological function. 3) The rat strain (Sprague Dawley) used was not ideal due to possibility of predisposed genetic factors and retinal susceptibility to light damage that could affect gene expression and confound the results of the study. These are inbred and albino animals and instead we could have used Long Evans which are outbred and pigmented rats and thus are more genetically robust and excessive light exposure resistant.

## 5 Conclusions

This study showed that traumatic physical injury caused by blast overpressure followed by head concussion led to degeneration of axons in the optic tracts, and this effect was greater in the animals fed DHA-deprived 1 en% LA diet. Inclusion of DHA in the HC diet leads to attenuation of blast-TBI induced disruption of neuronal plasticity and vascular function in the retina as well as suppressed ocular inflammatory signaling. Under psychological stress, having a dietary balance of ω-6 to ω-3 PUFAs as in the 1 en% LA group, is associated with an increase in the activity of neurotransmitters such as sympathomimetics (i.e., adrenaline, nor-adrenaline, and dopamine) and excitatory glutamate, and stimulation of calcium and endocannabinoid neuronal synapse signaling while suppressed pro-inflammatory molecular networks in the retina. Together our data advocate diets as a strong preemptive measure to combat the physical and psychological trauma. These findings will help fill the current gap in our understanding of how exposure to blast and psychological trauma events, in conjunction with improper nutrition, can lead to eye injuries and subsequent vision loss in warfighters and civilian population and will further address a knowledge platform for generation of new approaches to clinical diagnosis and care guidelines for TBI and acute traumatic stress.

## Data Availability

The original contributions presented in the study are publicly available. This data can be found here: https://www.ncbi.nlm.nih.gov/geo/, GSE243996.

## Author disclaimer

Material has been reviewed by the Walter Reed Army Institute of Research. There is no objection to its presentation and/or publication. The opinions or assertions contained herein are the private views of the author, and are not to be construed as official, or as reflecting true views of the Department of the Army or the Department of Defense. Research was conducted under an IACUC-approved animal use protocol in an AAALAC International - accredited facility with a Public Health Services Animal Welfare Assurance and in compliance with the Animal Welfare Act and other federal statutes and regulations relating to laboratory animals.
