# Supplementary material for: Impact of dietary changes on retinal neuronal plasticity in rodent models of physical and psychological trauma
Source: Front Genet. 2024 Sep 13;15:1373447. doi: 10.3389/fgene.2024.1373447 (PMC11427283; doi:10.3389/fgene.2024.1373447)

**Figure S1A:** Venn diagrams showing the levels of DEGs between different dietary conditions (p<0.05) on exposure to blast overpressure plus head concussion.


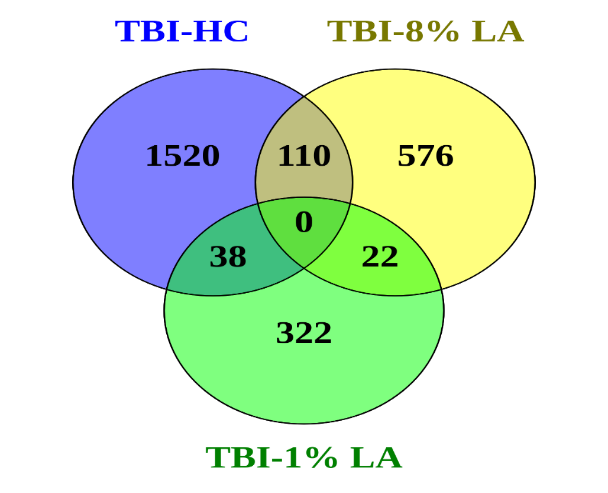


**Figure S1B:** Venn diagrams showing the levels of DEGs between different dietary conditions (p<0.05) on exposure to traumatic stress.


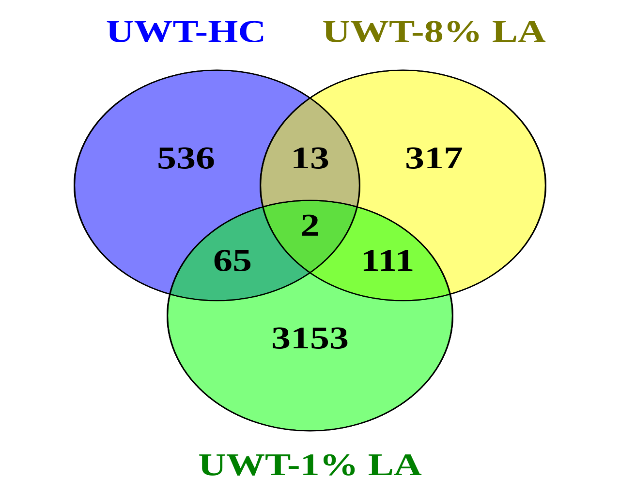


**Figure S2A:** Bar graph showing the influence of traumatic stress and DHA-deficient 1% LA diet on metabolic pathways in the retina.

**Figure S2B:** Molecular pathways dysregulated in the rat ocular tissue on exposure to traumatic stress in the 1% LA group.

**Figure S3:** The gene expression in endocannabinoid neuronal synapse pathway in the 1% LA group exposed to traumatic stress compared with sham 1% LA group. Endocannabinoids regulate synaptic function through retrograde signaling, autocrine signaling, and also indirectly by activating astrocytic receptors. Green/Red means down-/up-regulation. Blue network means inhibition while orange means activation (Left).

**UWT-1% LA vs Control-1% LA**


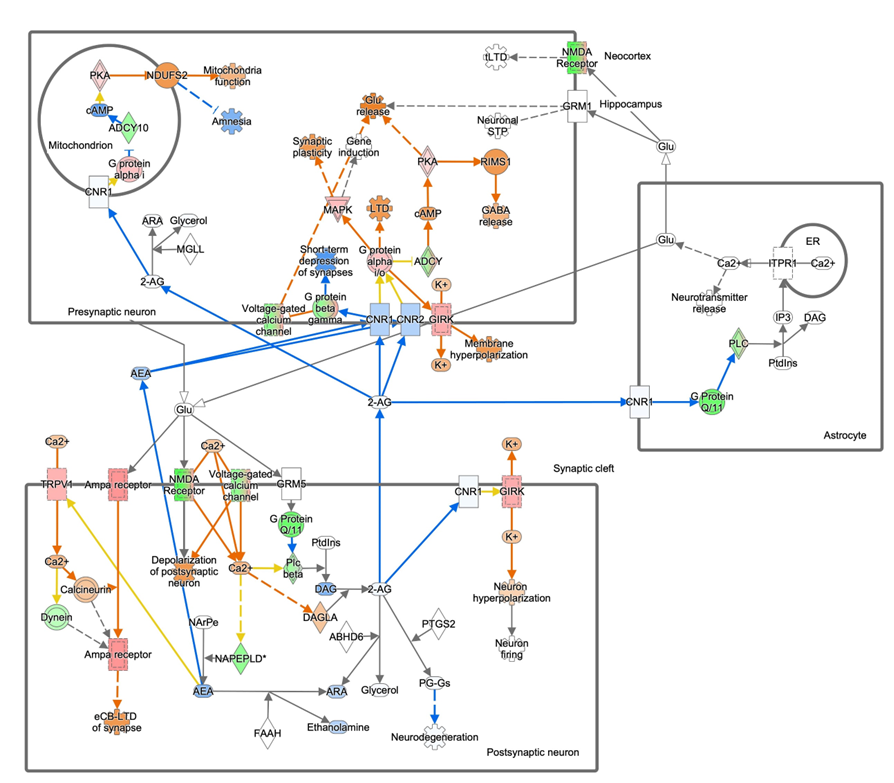

Supplement: Supplementary file 2 [file Table1.DOCX]
